# An Evaluation of Rebuilding Policies for U.S. Fisheries

**DOI:** 10.1371/journal.pone.0146278

**Published:** 2016-01-13

**Authors:** Ashleen Julia Benson, Andrew B. Cooper, Thomas R. Carruthers

**Affiliations:** 1 Canadian Fisheries Research Network, Biology Department, University of New Brunswick, Fredericton N.B., Canada; 2 School of Resource and Environmental Management, Simon Fraser University, Burnaby, B.C., Canada; 3 Fisheries Center, University of British Columbia, Vancouver, B.C., Canada; Aristotle University of Thessaloniki, GREECE

## Abstract

Rebuilding depleted fish populations is a priority of modern fisheries management. In the U.S., strong statutory mandates extend to both the goals and process by which stocks are to be rebuilt. However, the National Standard Guidelines that govern the implementation of the Magnuson-Stevens Fishery Conservation and Management Act may change to increase flexibility in rebuilding requirements. In this study we evaluate performance of the *status quo* approach to fish stock rebuilding in the United States against 3 alternatives that have been proposed to improve rebuilding outcomes. These alternatives either simplify the analytical requirements of rebuilding analyses or apply ‘best practices’ in fisheries management, thereby avoiding the need for rebuilding analyses altogether. We use a Management Strategy Evaluation framework to evaluate rebuilding options across 6 fish life history types and 5 possible real-world fishery scenarios that include options for stock assessment quality, multiple fleets, and the degree to which the stocks are overfished at the start of the analysis. We show that the *status quo* rebuilding plan and a harvest control rule that reduces harvest rates at low stock size generally achieve the best rebuilding outcomes across all life-history types and fishery scenarios. Both approaches constrain fishing in the short term, but achieve high catches in the medium and long term as stocks rebuild to productive levels. These results support a growing body of literature that indicates that efforts to end overfishing early pay off in the medium- to long-term with higher cumulative catches than the alternative.

## Introduction

Rebuilding depleted fish populations is a priority of modern fisheries management. Its importance is reflected in goals to rebuild fisheries to levels that can produce maximum sustainable yield (*MSY*) in the United Nations 2002 World Summit on Sustainable Development, the EU Common Fisheries Policy, and the legislative mandates of several nations including Australia and the United States (U.S.) [1, 2]. In the U.S., strong statutory mandates extend to both the goals and process by which stocks are to be rebuilt. For example, when a stock is designated as “overfished” (*B <* 0.5*B*_MSY_) a rebuilding plan that includes targets for both rebuilding time and exploitation rate must be established within 2 years.

The 1996 Sustainable Fisheries Act amendments to the Magnuson-Stevens Fishery Conservation and Management Act (MSFCMA) [3] specify that overfished stocks are to be rebuilt to biomass levels consistent with production of *MSY* in a period of time that is “as short as possible”, but not to exceed 10 years. Exceptions to this requirement can be made subject to species life history, environmental conditions, and international agreements. This legal mandate is operationalized using a framework recommended in the National Standard Guidelines of the National Marine Fisheries Service (NMFS) [4]. Populations that are predicted to be able to rebuild within 10 years in the absence of fishing are required to do so. Populations that cannot meet the 10-year time frame must be rebuilt within a period of time no longer than the minimum time to rebuild (e.g., rebuilding time with no fishing, *T*_MIN_), plus one generation time; this is called *T*_*MAX*_.

The 10-year time frame creates discrepancies in rebuilding approaches for short versus long-lived species and for lightly depleted versus heavily depleted stocks; these discrepancies are considered by some to be unfair and arbitrary [5]. For example, a stock for which *T*_MIN_ is predicted to be 9 years is required to rebuild within 10 years, even if that means a total moratorium on fishing, while a stock with *T*_MIN_ of 11 years would be permitted a longer time to rebuild (*T*_MAX_ = 11 years +1 generation time), which would likely allow for some fishing. The rigidity of the legislative structure surrounding the rebuilding framework has thus far restricted consideration of alternative analyses and management options, but NMFS has recently proposed revising the National Standard Guidelines [6] to allow greater flexibility in establishing the rebuilding timeline.

This study uses a management strategy evaluation (MSE) approach [7, 8] to evaluate the relative performance of alternative rebuilding strategies for U.S. fisheries. The analysis builds on the recommendations of Patrick and Cope [5] that evaluated different approaches to calculating *T*_MAX_, with the aim of avoiding the discontinuity in rebuilding plans arising from the 10-year requirement. According to their deterministic Graham-Schaefer model, rebuilding strategies based on simpler calculations of *T*_MAX_ generally met the 10-year rebuilding requirement and achieved more consistency between rebuilding plans. Our study extends their work by considering natural ecological variability and uncertainty in the management process, both of which can affect rebuilding timelines. Experience indicates that the rebuilding process can take many years, even decades [9], often due to continued exploitation that can be caused or exacerbated by ecological variability and management uncertainty [10, 11]. In contrast, success in rebuilding stock biomass is associated with early and sustained reductions in fishing mortality [11]. This study evaluates performance of “active” rebuilding policies against management approaches that do not explicitly attempt to achieve rebuilding, but which have been proposed as ‘best practices’ in the literature: the 40–10 rule [12] and 0.75*F*_MSY_ [13]. The latter policy is additionally one of the options considered in revisions to the National Standard Guidelines [6]. The goal of this study is to characterize performance of each management approach in terms of stock and fishery rebuilding, and to determine their relative performance across diverse life histories, assessment, and fishery scenarios.

## Materials and Methods

### Simulation approach

The MSE framework used in this study was originally developed by Carruthers et al. [14] to test performance of methods for setting catch limits in data-poor fisheries and runs in the R statistical platform [15]. It includes an operating model (OM) which simulates the “true” but unobserved dynamics of the fish population and fishery, an observation model which generates observed data subject to imprecision and bias, a stock assessment that uses the data to determine management recommendations (e.g. set target fishing mortality), and management policies that determine how the assessment will be used in either rebuilding plans or harvest control rules (HCR). The latter three processes are collectively referred to as management procedures (MP).

The structure of the biological component of the OM is identical to Carruthers et al. [14] and simulates age-structured dynamics for 6 life history types (mackerel (*Scombridae*), butterfish (*Stromateidae*), snapper (*Lutjanidae*), porgy (*Sparidae*), sole (*Pleuronectidae*), and rockfish (*Sebastidae*)). All parameter values can be found in Carruthers et al. [14] Appendix Table A.1. These are generic representations of real-world stocks. The fishery component of the OM is parameterized for either a single fleet or two fleets that can differ in terms of selectivity (the size of fish they target) and implementation error (how precisely and accurately the fishery attains the recommended catch). We ignored the spatial structure feature of the OM and simulated populations that are fully mixed throughout a single area. Each stock was fished for a 50-year period so that it was depleted to 6–12% of its unfished biomass (*B*_*0*_). This defines the starting conditions for a 90-year projection period in which future stock and fishery conditions are simulated with uncertainty under the various management procedures. This 90-year timeframe was chosen to accommodate the range of life histories and potential recovery times for the different species. We simulate the management procedures using the same data collection and delay-difference stock assessment model as in Carruthers et al. [14]. The delay-difference model uses information on catch (observed with error), fishing effort (a metric of fishing mortality rate), the stock-recruitment relationship, growth and natural mortality rates, and age-specific maturity and vulnerability to estimate stock status, current fishing mortality, and key management parameters *B*_0_, *B*_MSY_, *F*_MSY_. The assessment provides management advice every three years to approximate a typical assessment cycle [14].

Harvest advice during the rebuilding period is based on the assessment and either the rebuilding plans or HCRs described below. The realized catch deviates from the recommended catch (i.e., management implementation error) according to log-normally distributed errors, with or without bias, applied to the recommended catch. Every combination of life-history type and management procedure was simulated 300 times in order to produce a distribution of results for each combination. The structure and details of the management procedure component of the original MSE framework were modified to include two rebuilding plans and two management policies.

#### Rebuilding plans

The U.S. Sustainable Fisheries Act requires Rebuilding Plans for overfished stocks [4]. These plans typically include: (i) rebuilding analyses that estimate the time in which a population must be rebuilt to *B*_MSY_ (*T*_TARGET_, where *T*_MIN_ ≤ *T*_TARGET_ ≤ *T*_MAX_ depending on management objectives), and the fishing mortality rate that will allow the population to rebuild to *B*_MSY_ within *T*_TARGET_ (*F*_REB_), (ii) “rebuilding revision rules” to assess adequacy of progress toward rebuilding, and (iii) a process of modifying rebuilding plans based on assessed stock status [16]. However, there is no standard approach for conducting rebuilding analyses or revising rebuilding plans for U.S. fisheries [16, 17]. For this analysis we therefore simulate two simple versions that capture the main elements of rebuilding plans (e.g. calculate *T*_TARGET_ and *F*_REB_, and update plans periodically).

In year 1 of the projection period, the assessment model projects future stock conditions under no fishing to determine the time a stock will take to rebuild to *B*_MSY_ (*T*_MIN_). If *T*_MIN_ is less than 10 years, the stock must be rebuilt in 10 years. However, if the stock is unable to rebuild in 10 years in the absence of fishing, then the target year for rebuilding (*T*_TARGET_) may not be longer than *T*_MIN_ plus one mean generation time. We calculate mean generation time as *MGT* = $\sum_{a}^{A}L_{a}M_{a}$ where *L*_*a*_ and *M*_*a*_ represent survivorship and maturity at age for a given stock, respectively. This represents the *status quo* rebuilding approach [4]. The alternative rebuilding plan sets *T*_TARGET_ equal to twice *T*_MIN_, as proposed by Patrick and Cope [5] for consideration in the revisions to the National Standards Guidelines [6] and currently used by New Zealand’s Ministry of Primary Industry [18]. We refer to these as the *NMFS* and *2TMIN* management procedures, respectively. Both use the stock assessment model to conduct projections using a range of harvest rates and deterministic stock dynamics to identify the fishing mortality rate that allows the population to rebuild to *B*_MSY_ within *T*_TARGET_ (*F*_REB_). This differs from real-world rebuilding analyses, which use a stochastic stock projection and include a requirement that the probability of rebuilding under a given *F*_*REB*_ is ≥ 0.50 [16]. Similar to the HCRs described below, the estimation of *F*_REB_ is updated using the assessment model on a three-year cycle to ensure stocks will rebuild within *T*_TARGET_ that was identified in Year 1 of the projection period.

#### Harvest control rules

Management systems for U.S. fisheries commonly involve collecting data from the stock and fishery, conducting quantitative stock assessments to estimate stock status, and using this information in HCRs that translate management policies into management advice in terms of allowable catch or fishing mortality rates [12]. We apply two alternative HCRs in our simulated fishery management system. The first is the “40–10” rule that has been accepted by the Pacific Fishery Management Council [12]. If the stock is estimated to be above 40% of its unfished size (*B*_0_) the target catch is the population size multiplied by *F*_MSY_. If the stock is below 10% of its unfished size no catch is permitted. Between 10–40% of *B*_0_ the fishing mortality rate increases from 0 to *F*_MSY_. The second HCR we evaluate is 75% *F*_MSY_, which is commonly used in U.S. fisheries [19] and similar to a recommendation by Patrick and Cope [5]. Under this rule the target catch is simply the population size multiplied by *0*.*75F*_MSY_. We refer to these as the *40–10* and *0*.*75F*_*MSY*_ management procedures, respectively. Realized catches deviate from the rebuilding recommendations using the same log-normal implementation error as the rebuilding plans.

### Sensitivity analyses

The Baseline OM conditions represent the recovery dynamics of a single population subjected to a range of fishing conditions as determined by either rebuilding plans or HCRs that are updated on a 3-year cycle. These conditions apply to the six life-history types. Sensitivities of rebuilding plan and HCR performance were evaluated against a range of assumptions about the quality of the stock assessment, the rebuilding analysis, starting depletion, and fishery implementation of management recommendations. With the exception of starting depletion, these sensitivity runs encapsulate elements of the management system that can be modified to improve recovery outcomes. In each test, Baseline OM specifications are retained except for the specified revisions. The sensitivities include:

- Overestimate natural mortality (*M*) in the stock assessment, thereby assuming the stock is more productive than it is in the OM;
- Two fleet scenario in which the “true” fishery includes one fleet that has low implementation error and targets large fish (similar to a quota-based commercial fleet) and another fleet that has high implementation error with a bias towards higher-than-expected catches and targets smaller fish (similar to a recreational fleet).
- Calculate mean generation time (MGT) for the *NMFS* MP as the age at which 50% of that cohort is mature.
- During the 50-year period of fishing down, deplete the stocks to only 20–22% of the unfished biomass.

Additional sensitivity runs included high implementation error on the commercial fishery and an increased assessment and *F*_REB_ adjustment interval (5 year). These did not change simulation outcomes (in terms of the rank order of the four MPs) and are therefore not included in the results.

### Performance indicators

This analysis evaluates the relative performance of a suite of management procedures, and is not intended to identify the ‘best’ policy for a given species or fishery. The following performance indicators are used to reflect the general attributes of a rebuilding stock and fishery, and to compare performance of the MPs:

1. Probability that the alternative MPs rebuild the stock to *B*_MSY_ in at least as short a time as *NMFS* MP*;*
2. Probability that *NMFS* MP rebuilds the stock to *B*_*MSY*_ in at least as short a time as the alternative MPs;
3. Probability of rebuilding to *B*_MSY_ over short (10 year) and medium (40 year) time frames;
4. Average annual catch for short (10 year) and medium (40 year) time frames regardless of rebuilding status;
5. Average annual catch over the entire 90 years for those stocks that rebuilt to *B*_MSY_ within those 90 years.

## Results

The results of this simulation study indicate differences in the performance of the four MPs that persist across the sensitivity scenarios. Key outcomes are therefore presented using the Baseline simulations to illustrate the general patterns of outcomes across a range of life histories. Because the policy decision is whether to move away from the current *NMFS* rebuilding plan, and because the MSFCMA requires overfished stocks to rebuild in a period of time that is “as short as possible,” we first examine the probability that the alternative MPs rebuild stocks in a period of time that is at least as quick as *NMFS* (Performance Indicator 1). There is a greater than 50% chance that *2TMIN* will rebuild at least as quickly as *NMFS* for each of butterfish, snapper, porgy, and rockfish; however, there is a less than 50% chance *2TMIN* will rebuild at least as quickly for mackerel and sole (Table 1). Across all species, there is a 78–98% chance that the *40–10* MP will rebuild at least as quickly as *NMFS* (Table 1). The *0*.*75F*_MSY_ MP has a greater than 50% chance of rebuilding at least as quickly as *NMFS* for only mackerel, butterfish, and porgy (Table 1).

**Table 1:** Proportion of simulations in which (i) the three alternative MPs achieved as fast or faster rebuilding times than the *NMFS* MP (Performance indicator 1, top); and (ii) the *NMFS* MP achieved as fast or faster rebuilding times than the alternative MPs (Performance indicator 2, bottom) for the Baseline operating model scenario.

		2*T*_*MIN*_	40–10	0.75*F*_*MSY*_
Alternative	Mackerel	0.48	0.86	0.51
	Butterfish	0.95	0.98	0.94
	Snapper	0.53	0.95	0.25
	Porgy	0.57	0.89	0.56
	Sole	0.15	0.96	0.14
	Rockfish	0.53	0.78	0.41
*NMFS*	Mackerel	0.68	0.38	0.57
	Butterfish	0.94	0.58	0.67
	Snapper	0.79	0.44	0.81
	Porgy	0.54	0.38	0.47
	Sole	0.95	0.46	0.90
	Rockfish	0.57	0.36	0.61

Looking at only the probability that the alternative MPs do at least as well as *NMFS* hides the fact that in many cases the apparently good relative performance of the MP is due to a high percentage of times that the alternative MP and *NMFS* rebuild in the same amount of time (S1 Table). Another approach is to examine whether the current *NMFS* MP will rebuild stocks as fast or faster than an alternative (Performance Indicator 2). The *NMFS* MP has a 54–94% chance of rebuilding stocks as fast or faster than *2TMIN* across all species (Table 1). However, *NMFS* has a less than 50% chance rebuilding stocks as fast or faster than the *40–10* MP for all species except butterfish (Table 1). *NMFS* has a greater than 50% chance of rebuilding as fast or faster than *0*.*75F*_MSY_ for all species except porgy (Table 1).

**Fig 1:**
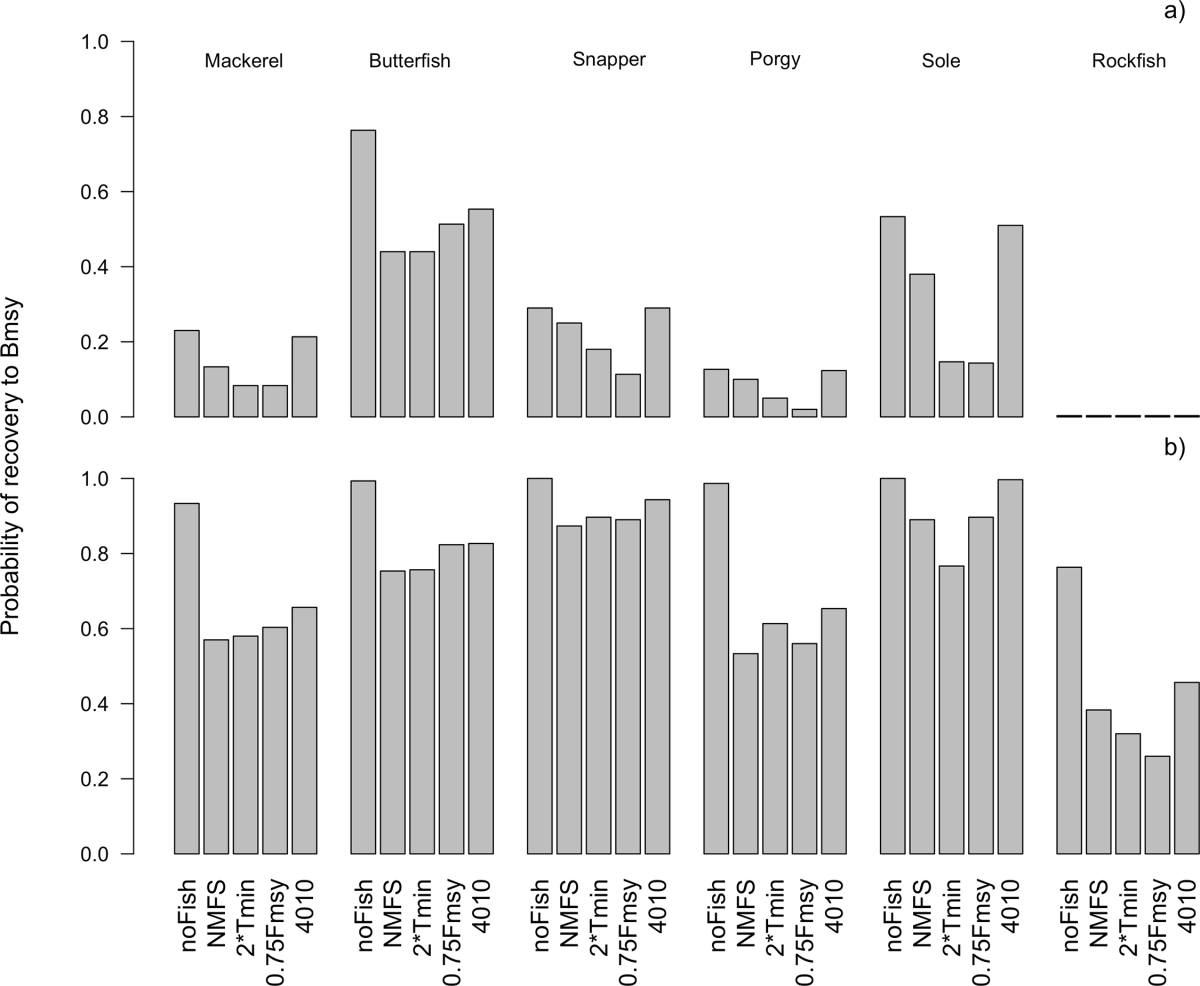
Probability of rebuilding to *B*_*MSY*_ in the first 10 years (a) and 40 years (b) of the simulation projection period for all species and management procedures using the Baseline model settings.

The probabilities above compare the current *NMFS* MP to alternative MPs across the entire 90 years of simulated rebuilding and management; however, these results do not necessarily scale when looking at the probability of rebuilding over short-term (10 years) and medium-term (40 years) time horizons (Performance Indicator 3). For all species the *40–10* MP demonstrates the highest probability of recovery to *B*_MSY_ over the short-term (10 years), nearing those realized under the ‘no fishing’ scenario (Fig 1A). With the exception of butterfish, the *NMFS* MP is consistently the second best MP evaluated over the short-term. The *2TMIN* MP did as well or better than the *0*.*75F*_*MSY*_, with the exception of butterfish. Over the medium-term, the *40–10* MP had the highest probability of rebuilding to *B*_*MSY*_ for all species (Fig 1B). The rank-ordering of *NMFS*, *2TMIN*, and 0.75F_MSY_ in the medium-term varied by life-history type, but the absolute probabilities of rebuilding to B_MSY_ were often quite similar (Fig 1B).

**Fig 2:**
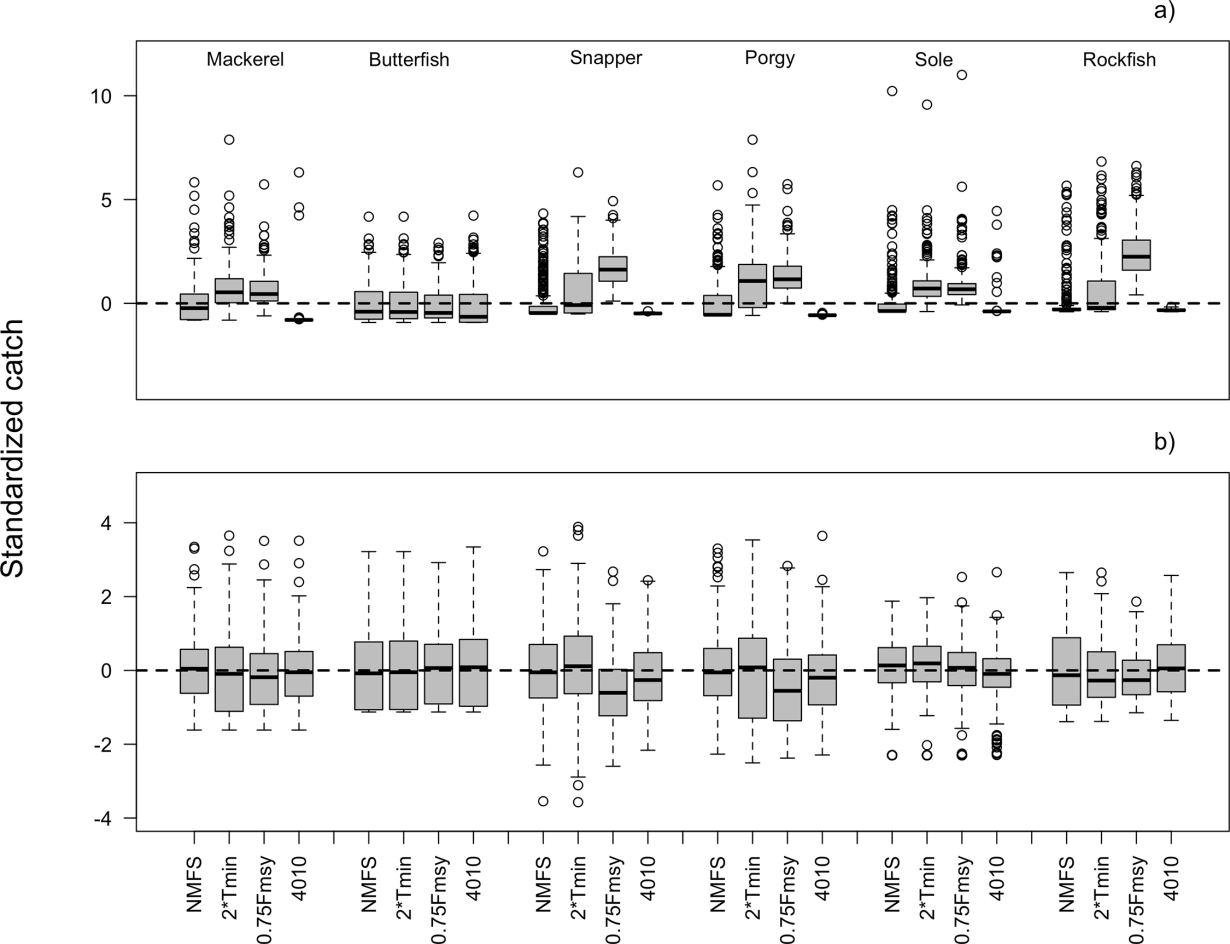
Average annual standardized catch in the first 10 years (a) and 40 years (b) of the simulation projection period for all species and management procedures using the Baseline model settings. **Catches are standardized against those realized under the *NMFS* management procedures (subtract the mean under *NMFS* and divide by the standard deviation under *NMFS*) to facilitate comparison across simulation scenarios and species.** Boxplots in (a) and (b) summarize the median, 25^th^, and 75^th^ percentiles; the whiskers correspond to the 5^th^ and 95^th^ percentiles, and the open circles are outliers.

**Fig 3:**
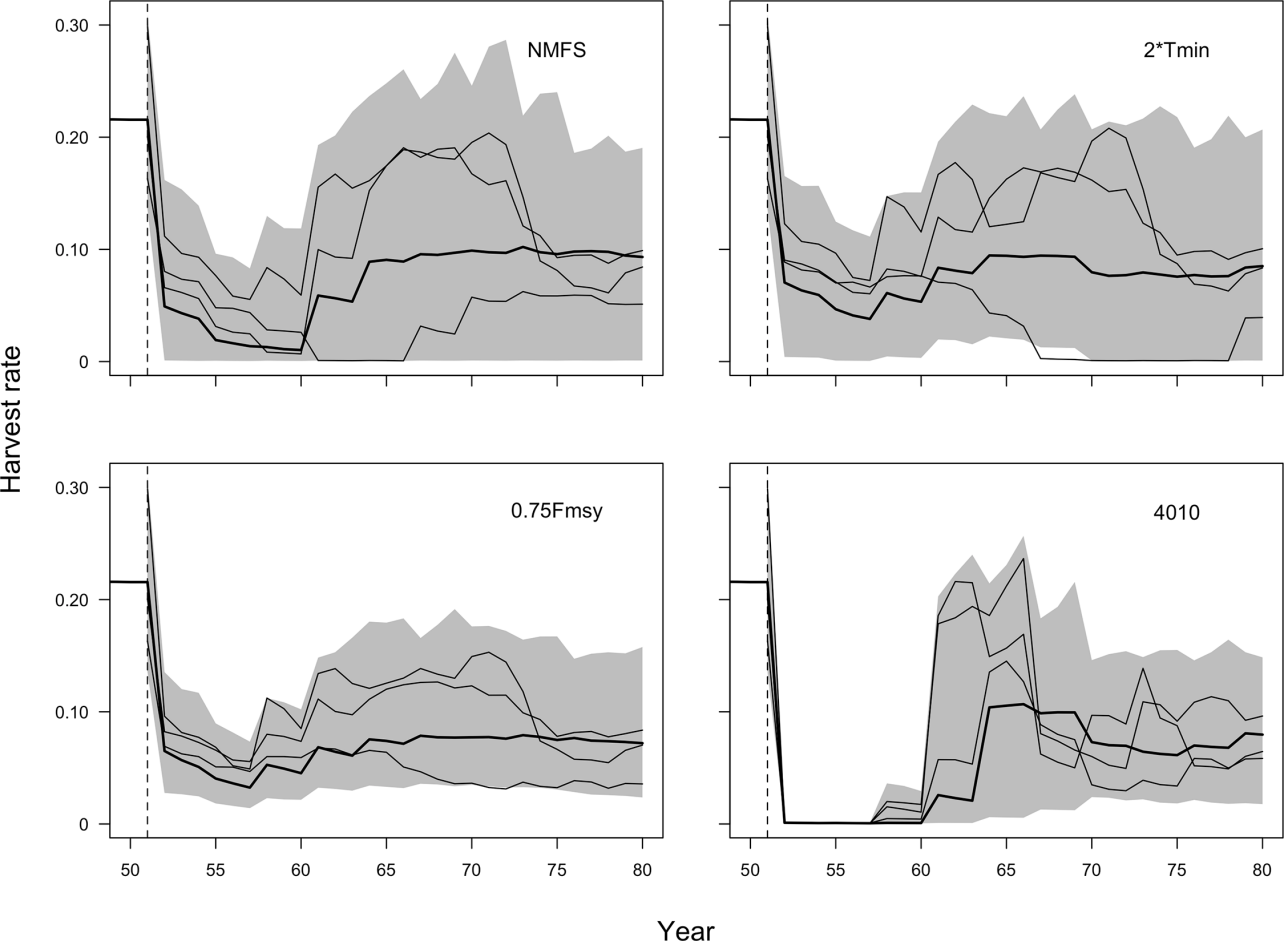
Example harvest rate envelopes using mackerel in simulation projection years 50–80 under each of the four management procedures. Simulation envelopes include the median (thick black line) and central 90% of harvest rates over 300 simulations (grey shaded region) and three individual simulation replicates (thin black lines).

Relative performance of the four MPs can be explained in part by differences in allowable catch (Performance Indicator 4). Higher catches in the short term translate to lower probabilities of rebuilding within the short term (compare Figs 1A and 2A) across all species. The *40–10* MP achieves the best rebuilding outcomes in the short term (Fig 1A) because it closes the fishery at low stock size and gradually increases harvest rates as the stock grows (compare short-term catches in Fig 2A and the harvest rate trajectories in Fig 3). The *NMFS* MP similarly achieves generally higher probabilities of rebuilding within 10 years (Fig 1A) by setting low catches in the short-term (Fig 2A) due to the requirement to rebuild stocks within 10 years if it is possible to do so in the absence of fishing. In the medium-term, however, this relationship breaks down, and except in the cases of porgy and snapper, the catch is quite comparable across MPs (Fig 2B). MPs with higher probability of rebuilding within 40 years (Fig 1B) do not necessarily have lower average catches over those 40 years (Fig 2B). For example, even though the *40–10* MP has the highest probability of recovering within 40 years across all species (Fig 1B), the average catch under the *40–10* MP is comparable to the catch under the other MPs (Fig 2B).

**Fig 4:**
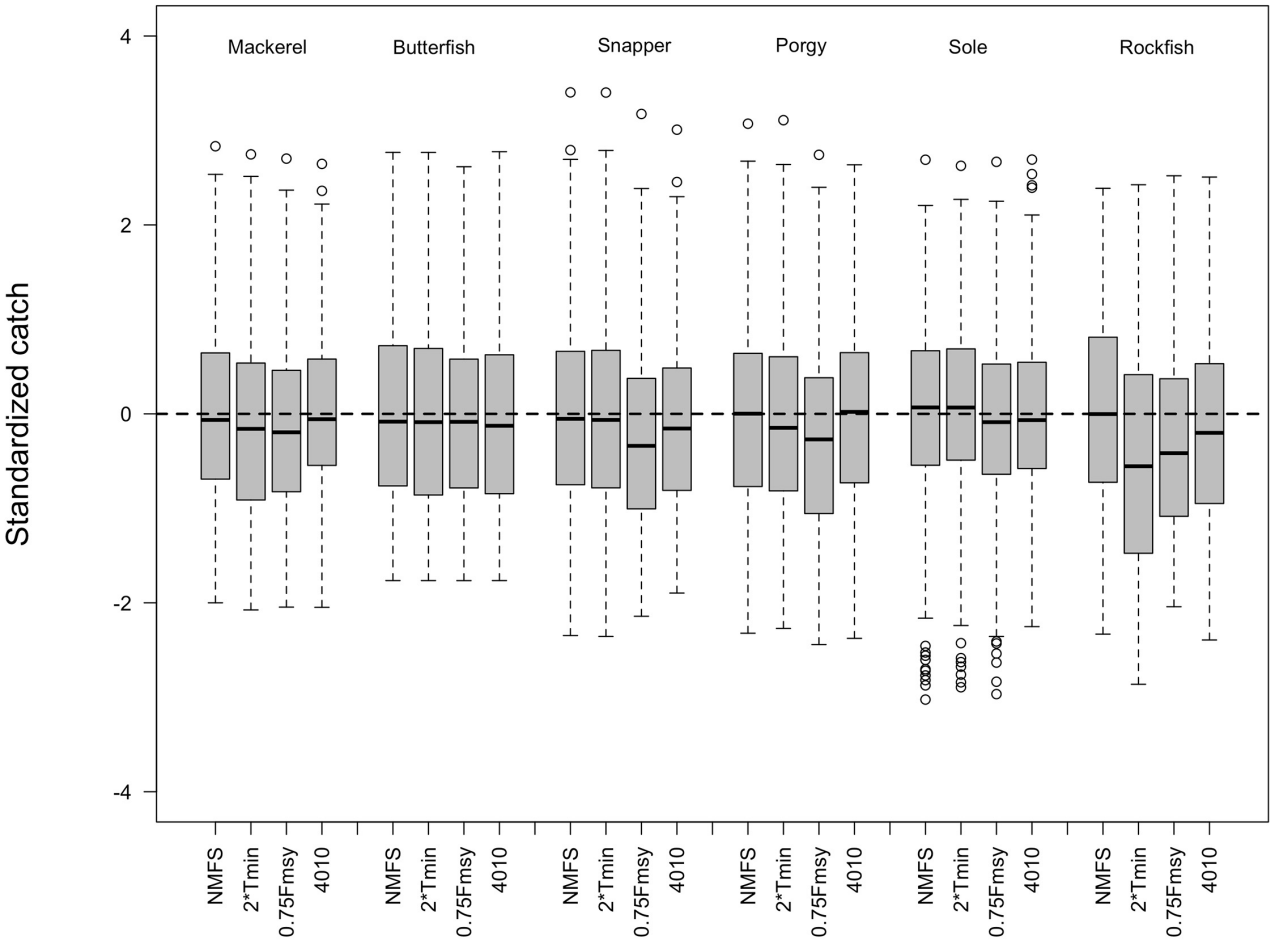
Average annual standardized catch for simulations in which the stock rebuilt to *B*_*MSY*_ within the 90-year simulation projection period. Plot includes all species and management procedures using the Baseline model settings. Boxplots summarize the median, 25^th^, and 75^th^ percentiles; the whiskers correspond to the 5^th^ and 95^th^ percentiles, and the open circles are outliers.

The inconsistent patterns in average catch across MPs in the medium term (Fig 2B) disappear when looking at the average catch across the full 90 years for those stocks that rebuilt sometime during those 90 years (Performance Indicator 5). The *NMFS* MP had either the highest or nearly the highest median (across simulations) of the average catch during the 90 years for all species (Fig 4). The rank-ordering of the median catches (across simulations) for the other MPs differed by life history, but 0.75F_MSY_ tended to be equal to or lower than the others with the exception of rockfish (Fig 4).

The absolute values of the performance indicators changed with the sensitivity runs, but in general the rank-ordering of the MPs remained stable (Table 2, S2 and S3 Tables). The starting depletion sensitivity is the exception. When stocks were depleted to only 20–22% of the unfished biomass during the 50-year period of fishing down, the differences described above became less pronounced, and the performance in terms of probability of rebuilding and catch became more similar across MPs (Table 2, S2, S3, and S4 Tables).

**Table 2:** Proportion of simulations in which (i) the three alternative MPs achieved as fast or faster rebuilding times than the *NMFS* MP (Performance indicator 1, top); and (ii) the *NMFS* MP achieved as fast or faster rebuilding times than the alternative MPs (Performance indicator 2, bottom) for the starting depletion sensitivity scenario.

		2*T*_*MIN*_	40–10	0.75*F*_*MSY*_
Alternative	Mackerel	0.73	0.89	0.49
	Butterfish	0.98	0.99	0.97
	Snapper	0.74	0.97	0.44
	Porgy	0.91	0.84	0.35
	Sole	0.81	0.95	0.46
	Rockfish	0.86	0.79	0.31
*NMFS*	Mackerel	0.77	0.50	0.66
	Butterfish	0.96	0.77	0.74
	Snapper	0.95	0.44	0.85
	Porgy	0.92	0.80	0.93
	Sole	0.83	0.45	0.68
	Rockfish	0.92	0.75	0.74

To further explore the patterns observed in the probability of *NMFS* rebuilding as fast or faster than the alternative MPs and the probability of the alternative MPs rebuilding as fast or faster than *NMFS*, we calculated correlations between these probabilities and the parameters specified in the operating model. The probability of *NMFS* rebuilding as fast or faster than the *2TMIN* MP increased as the maximum value for the steepness of the stock-recruit relationship increased (r = 0.90, p = 0.02). The probability of *NMFS* rebuilding as fast or faster than the *0*.*75F*_*MSY*_ MP increased as both the minimum steepness and maximum steepness of the stock-recruit relationship increased (r = 0.92, p = 0.001; r = 0.90, p = 0.02, respectively). The probability of *NMFS* rebuilding as fast or faster than the *40–10* MP increased as minimum and maximum values for natural mortality increased (r = 0.86, p = 0.03; r = 0.85, p = 0.03). The probability of the *2TMIN* MP rebuilding as fast or faster than *NMFS* increased as the minimum and maximum values for the stochastic process error increased (r = 0.86, p = 0.03; r = 0.96, p = 0.002). The probability of the *0*.*75F*_*MSY*_ MP rebuilding as fast or faster than *NMFS* increased with increasing values for the minimum and maximum values for natural mortality (r = 0.85, p = 0.03; r = 0.85, p = 0.03) along with the minimum and maximum values for the stochastic process error (r = 0.81, p = 0.05; r = 0.84, p = 0.04). The probability of the *40–10* MP rebuilding as fast or faster than *NMFS* was not correlated with any life history parameters.

To examine the importance of the 10-year rebuilding requirement in the *NMFS* MP (if the stock can rebuild within 10 years in the absence of fishing, then the maximum rebuilding time is 10 years), we compared the rebuilding times across MPs for those stocks that rebuilt under the *NMFS* MP within 10 years and those that did not. When the *NMFS* MP rebuilt a stock within 10 years, both the *2TMIN* and *0*.*75F*_*MSY*_ MPs took as long (in the case of butterfish) or longer than the *NMFS* MP (S1A Fig). The *40–10* MP, however, rebuilt these stocks at an even faster rate than *NMFS* (S1A Fig). The shorter rebuilding times for *NMFS* relative to *2TMIN* and *0*.*75F*_*MSY*_ are not nearly as consistent for those stocks that took longer than 10 years for *NMFS* to rebuild, with *NMFS* sometimes taking far longer to rebuild (S1B Fig). Those stocks that failed to rebuild were assigned a rebuilding time of 100 years, and in some scenarios this constitutes more than 25% of the simulations (e.g., the *NMFS* MP for mackerel and all MPs for rockfish). Because the boxplots examine percentiles, results are robust to the specific value of the rebuilding time used for those stocks that did not rebuild within 90 years.

## Discussion

The U.S. has a strong legislative mandate with clear requirements to end overfishing and rebuild depleted fish stocks. However, broad-scale evaluations of progress toward rebuilding show that the effectiveness of rebuilding plans is mixed [10, 11, 20]. Two factors have been proposed as limiting rebuilding progress. The first is uncertainty in the scientific process of establishing rebuilding targets and exploitation rates that arises from a lack of data, natural ecological variability, and modeling of inherently dynamic processes [11, 20]. The second reason is an inability or unwillingness on the part of fisheries managers to restrict fishing mortality early in the rebuilding process [10, 11].

Regarding the first issue, the current *NMFS* rebuilding plan is analytically intensive, relying on biomass-based targets and reference points for estimating *B*_MSY_, *T*_MIN_, *T*_MAX_, and *F*_REB_, as well as to periodically assess stock status relative to the target. Scientific uncertainty therefore impacts the initial designation of ‘overfished’, the establishment of a rebuilding plan, and the assessment of progress toward rebuilding. Proposals aimed at reducing scientific uncertainty include focusing on fishing mortality targets rather than timelines for achieving biomass targets, simplifying the calculation of *T*_MAX_, or using a precautionary harvest control rule to both reduce the probability of driving stocks to overfished levels and to rebuild depleted stocks [5, 20, 21]. This analysis investigated each of these options, with the *a priori* expectation that simpler procedures would outperform the *status quo NMFS* MP across the range of life histories, and process, observation, and implementation errors that were represented by the simulation model. Furthermore, previous authors suggested that *F-*based reference points (and presumably, management strategies based on them) are more robust to uncertainty than biomass-based reference points for both rebuilding and fishery management [20]. It was therefore surprising that the *NMFS* MP often performed comparably or better than the *0*.*75F*_MSY_ and *2TMIN* MPs. The *NMFS* MP often had a higher probability of rebuilding faster than or equal to either the *0*.*75F*_MSY_ and *2TMIN* MPs than the alternatives had for rebuilding faster than or equal to *NMFS*. (Table 1, bottom vs. top, and S3 Table). Similarly, the probability of rebuilding within short and medium time frames was often comparable or higher for *NMFS* than for either the *0*.*75F*_MSY_ and *2TMIN* MPs (Fig 1). The *NMFS* requirement that if a stock can be rebuilt in 10 years in the absence of fishing it must be rebuilt within 10 years is likely one of the reasons for its better performance relative to the *0*.*75F*_MSY_ and *2TMIN* MPs (S1A Fig). Given the MSFCMA requirement that overfished stocks be rebuilt in a period of time that is “as short as possible,” it would be difficult to justify switching to either the *0*.*75F*_MSY_ and *2TMIN* MPs as modeled here. However, given the variable responses across the generic life histories represented in this analysis, management strategy evaluations for specific fisheries might justify these or alternative approaches.

The *40–10* MP achieved similar or better outcomes to the *NMFS* MP, while requiring relatively fewer calculations for implementation. This approach avoids the complexities of the rebuilding plan forecasts of future rebuilding trajectories and timelines, but its key benefit in terms of rebuilding is that it constrains fishing at low stock size (Figs 1, 2B, 3). The *NMFS* MP similarly constrains fishing in the short term if the stock can be rebuilt in 10 years, but with less conservative catches.

While the MSFCMA is largely concerned with rebuilding stocks as quickly as possible, one cannot ignore the effect the MPs have on short, medium, and long-term catches. As stated above, both the *NMFS* and *40–10* MPs achieve their faster rebuilding by constraining catches early in the rebuilding timeline. However, medium and long-term catches are similar across all MPs. Once a stock is rebuilt (or above 40% of *B*_*0*_ for the *40–10* MP), the fishing rate is set to *F*_*MSY*_, thus allowing higher fishing on this larger stock and balancing the foregone catch of the early years for the *NMFS* and *40–10* MPs (not accounting for discount rates). These results are similar to those of Shertzer and Prager [22], who showed that ending overfishing earlier rather than later produced higher cumulative catches. It is important to note, though, that the *40–10* MP was originally conceived as a management procedure to prevent overfishing rather than an approach for rebuilding stocks. The severe limits to catch in the short term with the *40–10* MP are because of the severely depleted state of the stocks (6–12% of the unfished state). When stocks are depleted to only 20–22% of the unfished state, the *40–10* MP has comparable (0.39 vs. 0.40 for rockfish) or higher probabilities of rebuilding in the short term than the *NMFS* MP, yet has higher average catch than *NMFS* in the short term for all species except butterfish and sole (S2 Table).

We did not conduct a comprehensive examination of the uncertainties inherent in each procedure tested, however, changes in assessment quality (compare the Baseline and High *M* scenarios in S2 Table) and frequency (results not shown) did achieve small changes in the performance of a given procedure, but did not change the rank order of outcomes. This suggests that scientific uncertainty can impact management outcomes, but reducing the scientific *complexity* of rebuilding or management plans by either reducing the number of uncertain parameters (mean generation time in the case of *2TMIN*) or focusing on *F-*based approaches does not appear to consistently improve rebuilding outcomes. However, management strategy evaluations tailored for specific fisheries may prove that these alternative rebuilding approaches are more successful.

This analysis builds on a previous study by Patrick and Cope [5] which found that that an average overfished stock could rebuild in 10 years under both the *2TMIN* and 0.75*F*_MSY_ MPs. Our analysis does not support their conclusion, and finds the *status quo* MP and the *40–10* MP achieve better outcomes overall. The difference between the results is likely due to the markedly different analytical approaches. Whereas Patrick and Cope [5] relied on a deterministic model to evaluate the alternative rebuilding options, we used an MSE approach that accounted for uncertainty in the natural ecological processes, data collection, stock assessment, and implementation of management recommendations. This approach is considered a ‘best practice’ in fisheries management that identifies management strategies (or rebuilding plans) that are robust to uncertainty [23]. Our results are similar to Babcock et al. [24] who found that an MP in line with the *40–10* MP rebuilt stocks either the fastest or second fastest of all the MPs they considered and the current *NMFS* MP was the only one to ever rebuild stocks faster. We emphasize, however, that in other countries with different policy mandates, data availability, and less control over fishing effort other methods may be more robust and appropriate than those tested here. Babcock et al. [24] used a stochastic projection model, which incorporated more uncertainty than Patrick and Cope [5], but did not model the full data collection, assessment, and management feedback loops. While our overall approach accounts for more uncertainty than Patrick and Cope [5] and Babcock et al. [24], our analysis does not account for all potential sources of uncertainty. For example, the failure of some stocks to rebuild as expected has been attributed to long-term shifts in population productivity or ecosystem structure [10, 25]. More recently, research has suggested that the dynamics of many fish populations may be characterized by abrupt and then lasting shifts in productivity that may not be accounted for by the simulation framework presented here [26]. Future rebuilding analyses could aim to address this issue and evaluate rebuilding strategies using ecosystem operating models or single species operating models that simulate longer-term regime shifts.

## Supporting Information

S1 Fig Summary of recovery time by management procedure and species for (a) Baseline simulations in which the *NMFS* MP recovered in 10 years or less, and (b) Baseline simulations in which the *NMFS* MP recovered in greater than 10 years. Recovery time in (b) was set to 100 years for simulations that failed to recover to illustrate the range of realized outcomes. Boxplots in (a) and (b) summarize the median, 25^th^, and 75^th^ percentiles; the whiskers correspond to the 5^th^ and 95^th^ percentiles, and the open circles are outliers.
(DOCX)

S1 Table Proportion of simulations in which (i) the *NMFS* MP rebuilt faster than the *2TMIN* MP, the *NMFS* and *2TMIN* MPs rebuilt in the same amount of time, and the *2TMIN* MP rebuilt faster than the *NMFS* MP (top); (ii) the *NMFS* MP rebuilt faster than the *40–10* MP, the *NMFS* and *40–10* MPs rebuilt in the same amount of time, and the *40–10* MP rebuilt faster than the *NMFS* MP (middle); and (iii) the *NMFS* MP rebuilt faster than the *0*.*75F*_*MSY*_ MP, the *NMFS* and *0*.*75F*_*MSY*_ MPs rebuilt in the same amount of time, and the *0*.*75F*_*MSY*_ MP rebuilt faster than the *NMFS* MP (bottom). (DOCX)

S2 Table Management procedure performance for all operating model scenarios and sensitivities, including species, stock assessment and rebuilding analyses (high M and MGT, respectively), starting depletion (initial *D*), and fishing fleets. P_REC_ is the probability of rebuilding to *B*_*MSY*_ within 10 or 40 years; catch is the median annual catch measured over the same time periods. The 2-fleet sensitivity scenario applies only to species that are targeted by both commercial and recreational fisheries (Snapper, Red porgy, and Canary rockfish).
(DOCX)

S3 Table Proportion of simulations in which (i) the three alternative MPs achieved as fast or faster rebuilding times than the *NMFS* rebuilding plan (Performance indicator 1, top); and (ii) the *NMFS* rebuilding plan achieved as fast or faster rebuilding times than the alternative MPs (Performance indicator 2, bottom). (DOCX)

S4 Table When stocks were depleted to only 20–22% of the unfished biomass during the 50-year period of fishing down, proportion of simulations in which (i) the *NMFS* MP rebuilt faster than the *2TMIN* MP, the *NMFS* and *2TMIN* MPs rebuilt in the same amount of time, and the *2TMIN* MP rebuilt faster than the *NMFS* MP (top); (ii) the *NMFS* MP rebuilt faster than the *40–10* MP, the *NMFS* and *40–10* MPs rebuilt in the same amount of time, and the *40–10* MP rebuilt faster than the *NMFS* MP (middle); and (iii) the *NMFS* MP rebuilt faster than the *0*.*75F*_*MSY*_ MP, the *NMFS* and *0*.*75F*_*MSY*_ MPs rebuilt in the same amount of time, and the *0*.*75F*_*MSY*_ MP rebuilt faster than the *NMFS* MP (bottom). Compare to S1 Table.
(DOCX)
