# Supplementary figures and images for: An Evaluation of Rebuilding Policies for U.S. Fisheries
Source: PLoS One. 2016 Jan 13;11(1):e0146278. doi: 10.1371/journal.pone.0146278 (PMC4711967; doi:10.1371/journal.pone.0146278)

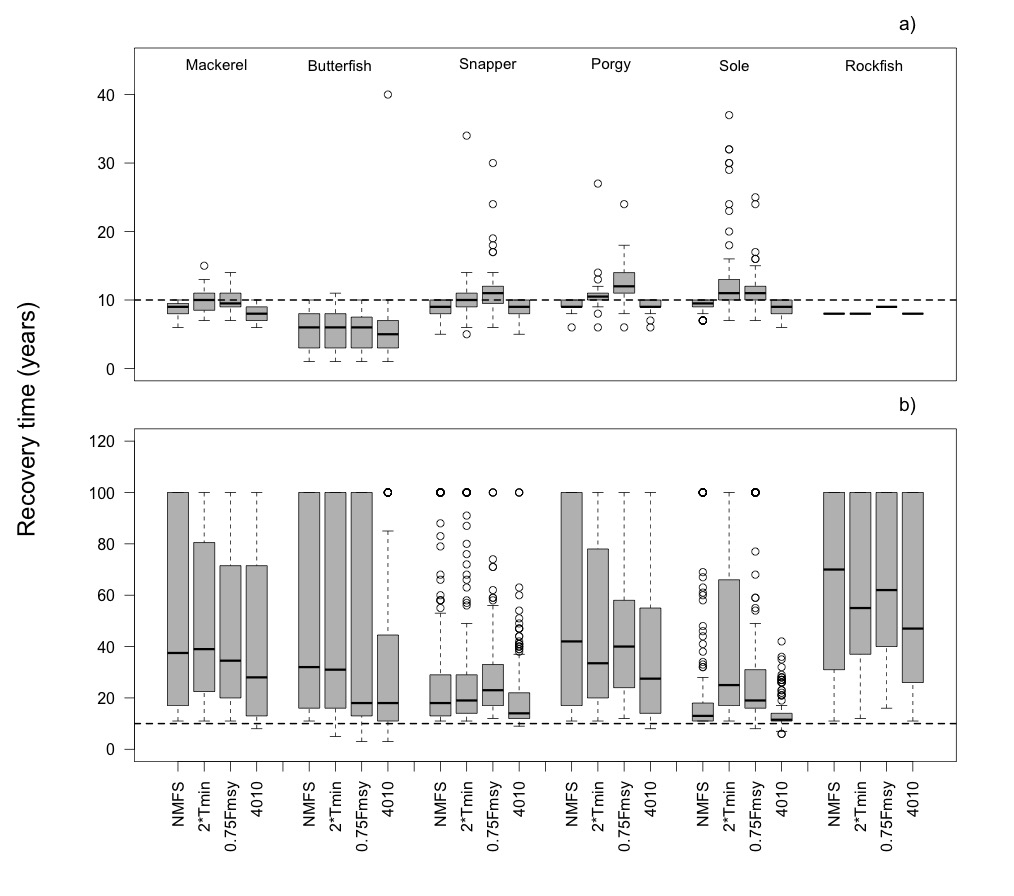


S1 Fig.

Supplement: S1 Fig — Recovery time in (b) was set to 100 years for simulations that failed to recover to illustrate the range of realized outcomes. Boxplots in (a) and (b) summarize the median, 25th, and 75th percentiles; the whiskers correspond to the 5th and 95th percentiles, and the open circles are outliers. (DOCX) [file pone.0146278.s001.docx]
