# Supplementary material for: An Evaluation of Rebuilding Policies for U.S. Fisheries
Source: PLoS One. 2016 Jan 13;11(1):e0146278. doi: 10.1371/journal.pone.0146278 (PMC4711967; doi:10.1371/journal.pone.0146278)
Supplement: S1 Table — (DOCX) [file pone.0146278.s002.docx]

S1 Table.

|  | **NMFS Faster** | **Equal** | **2TMIN Faster** |
| --- | --- | --- | --- |
| Mackerel | 0.52 | 0.16 | 0.32 |
| Butterfish | 0.05 | 0.89 | 0.06 |
| Snapper | 0.47 | 0.32 | 0.21 |
| Porgy | 0.43 | 0.11 | 0.46 |
| Sole | 0.85 | 0.09 | 0.05 |
| Rockfish | 0.47 | 0.10 | 0.43 |
|  |  |  |  |
|  | **NMFS Faster** | **Equal** | **40-10 Faster** |
| Mackerel | 0.14 | 0.24 | 0.62 |
| Butterfish | 0.02 | 0.56 | 0.42 |
| Snapper | 0.05 | 0.38 | 0.56 |
| Porgy | 0.11 | 0.26 | 0.62 |
| Sole | 0.04 | 0.42 | 0.54 |
| Rockfish | 0.22 | 0.14 | 0.64 |
|  |  |  |  |
|  | **NMFS Faster** | **Equal** | **0.75Fmsy Faster** |
| Mackerel | 0.49 | 0.09 | 0.43 |
| Butterfish | 0.06 | 0.61 | 0.33 |
| Snapper | 0.75 | 0.06 | 0.19 |
| Porgy | 0.44 | 0.02 | 0.53 |
| Sole | 0.86 | 0.04 | 0.10 |
| Rockfish | 0.59 | 0.02 | 0.39 |
