# Supplementary material for: An Evaluation of Rebuilding Policies for U.S. Fisheries
Source: PLoS One. 2016 Jan 13;11(1):e0146278. doi: 10.1371/journal.pone.0146278 (PMC4711967; doi:10.1371/journal.pone.0146278)
Supplement: S2 Table — PREC is the probability of rebuilding to BMSY within 10 or 40 years; catch is the median annual catch measured over the same time periods. The 2-fleet sensitivity scenario applies only to species that are targeted by both commercial and recreational fisheries (Snapper, Red porgy, and Canary rockfish). (DOCX) [file pone.0146278.s003.docx]

S2 Table.

|  |  | **Baseline** | | | | **High *M*** | | | | **MGT** | | | | **Initial *D*** | | | | **2 Fleet** | | | |
| --- | --- | --- | --- | --- | --- | --- | --- | --- | --- | --- | --- | --- | --- | --- | --- | --- | --- | --- | --- | --- | --- |
|  |  | **P_REC_** | | **Catch** | | **P_REC_** | | **Catch** | | **P_REC_** | | **Catch** | | **P_REC_** | | **Catch** | | **P_REC_** | | **Catch** | |
|  |  | **10y** | **40y** | **10y** | **40y** | **10y** | **40y** | **10y** | **40y** | **10y** | **40y** | **10y** | **40y** | **10y** | **40y** | **10y** | **40y** | **10y** | **40y** | **10y** | **40y** |
| **Mackerel** | **NoFish** | 0.23 | 0.93 | -- | -- | 0.24 | 0.90 | -- | -- | 0.18 | 0.93 | -- | -- | 0.65 | 0.99 | -- | -- | -- | -- | -- | -- |
|  | **NMFS** | 0.13 | 0.57 | 0.51 | 6.14 | 0.09 | 0.43 | 1.03 | 4.78 | 0.09 | 0.58 | 0.30 | 6.18 | 0.50 | 0.76 | 0.14 | 7.71 | -- | -- | -- | -- |
|  | **2Tmin** | 0.08 | 0.58 | 1.17 | 4.71 | 0.04 | 0.46 | 1.54 | 3.86 | 0.03 | 0.57 | 1.21 | 4.29 | 0.42 | 0.75 | 1.66 | 7.56 | -- | -- | -- | -- |
|  | **.75FMSY** | 0.08 | 0.60 | 1.10 | 5.11 | 0.06 | 0.52 | 1.34 | 4.77 | 0.03 | 0.61 | 1.15 | 4.94 | 0.36 | 0.82 | 2.64 | 6.95 | -- | -- | -- | -- |
|  | **4010** | 0.21 | 0.66 | 0.02 | 5.56 | 0.21 | 0.49 | 0.03 | 4.57 | 0.16 | 0.65 | 0.02 | 5.74 | 0.62 | 0.84 | 0.47 | 7.46 | -- | -- | -- | -- |
|  |  |  |  |  |  |  |  |  |  |  |  |  |  |  |  |  |  |  |  |  |  |
| **Butterfish** | **NoFish** | 0.76 | 0.99 | -- | -- | 0.78 | 0.99 | -- | -- | 0.78 | 1.00 | -- | -- | 0.88 | 1.00 | -- | -- | -- | -- | -- | -- |
|  | **NMFS** | 0.44 | 0.75 | 0.41 | 0.93 | 0.46 | 0.72 | 0.56 | 1.04 | 0.46 | 0.75 | 0.58 | 0.93 | 0.64 | 0.83 | 0.85 | 1.00 | -- | -- | -- | -- |
|  | **2Tmin** | 0.44 | 0.76 | 0.40 | 0.93 | 0.46 | 0.73 | 0.56 | 1.04 | 0.46 | 0.74 | 0.58 | 0.91 | 0.64 | 0.83 | 0.85 | 1.05 | -- | -- | -- | -- |
|  | **.75FMSY** | 0.51 | 0.82 | 0.36 | 1.05 | 0.51 | 0.80 | 0.54 | 1.01 | 0.51 | 0.84 | 0.51 | 1.01 | 0.70 | 0.89 | 0.75 | 1.07 | -- | -- | -- | -- |
|  | **4010** | 0.55 | 0.83 | 0.21 | 1.09 | 0.53 | 0.80 | 0.46 | 1.08 | 0.53 | 0.85 | 0.37 | 0.99 | 0.69 | 0.91 | 0.64 | 1.12 | -- | -- | -- | -- |
|  |  |  |  |  |  |  |  |  |  |  |  |  |  |  |  |  |  |  |  |  |  |
| **Sole** | **NoFish** | 0.53 | 1.00 | -- | -- | 0.51 | 1.00 | -- | -- | 0.51 | 1.00 | -- | -- | 0.99 | 1.00 | -- | -- | -- | -- | -- | -- |
|  | **NMFS** | 0.38 | 0.89 | 0.08 | 13.83 | 0.27 | 0.79 | 0.45 | 13.89 | 0.38 | 0.91 | 0.08 | 13.52 | 0.89 | 0.97 | 1.76 | 14.96 | -- | -- | -- | -- |
|  | **2Tmin** | 0.15 | 0.77 | 2.34 | 12.92 | 0.10 | 0.60 | 2.72 | 12.79 | 0.16 | 0.78 | 2.24 | 12.64 | 0.83 | 0.96 | 2.74 | 15.07 | -- | -- | -- | -- |
|  | **.75FMSY** | 0.14 | 0.90 | 2.27 | 12.40 | 0.13 | 0.83 | 2.33 | 13.26 | 0.16 | 0.90 | 2.17 | 12.15 | 0.79 | 0.99 | 4.83 | 14.45 | -- | -- | -- | -- |
|  | **4010** | 0.51 | 1.00 | 0.04 | 11.40 | 0.50 | 0.94 | 0.04 | 12.29 | 0.49 | 0.99 | 0.04 | 11.28 | 0.97 | 1.00 | 0.48 | 13.65 | -- | -- | -- | -- |
|  |  |  |  |  |  |  |  |  |  |  |  |  |  |  |  |  |  |  |  |  |  |
| **Snapper** | **NoFish** | 0.29 | 1.00 | -- | -- | 0.31 | 1.00 | -- | -- | 0.28 | 1.00 | -- | -- | 0.97 | 1.00 | -- | -- | 0.43 | 1.00 | -- | -- |
|  | **NMFS** | 0.25 | 0.87 | 0.11 | 13.15 | 0.27 | 0.86 | 0.18 | 12.23 | 0.24 | 0.89 | 0.10 | 13.73 | 0.95 | 0.97 | 0.21 | 16.62 | 0.39 | 0.91 | 0.11 | 14.99 |
|  | **2Tmin** | 0.18 | 0.90 | 0.60 | 12.94 | 0.17 | 0.90 | 2.41 | 11.61 | 0.16 | 0.88 | 0.87 | 12.54 | 0.94 | 0.98 | 0.22 | 16.63 | 0.30 | 0.89 | 0.21 | 14.83 |
|  | **.75FMSY** | 0.11 | 0.89 | 2.80 | 10.93 | 0.11 | 0.82 | 3.09 | 10.64 | 0.09 | 0.88 | 2.61 | 10.33 | 0.79 | 0.99 | 4.70 | 14.62 | 0.19 | 0.90 | 3.13 | 12.87 |
|  | **4010** | 0.29 | 0.94 | 0.09 | 12.87 | 0.31 | 0.87 | 0.08 | 13.36 | 0.28 | 0.96 | 0.08 | 12.25 | 0.96 | 1.00 | 0.81 | 14.65 | 0.43 | 0.97 | 0.08 | 13.87 |
|  |  |  |  |  |  |  |  |  |  |  |  |  |  |  |  |  |  |  |  |  |  |
| **Porgy** | **NoFish** | 0.13 | 0.99 | -- | -- | 0.12 | 0.99 | -- | -- | 0.10 | 0.99 | -- | -- | 0.76 | 1.00 | -- | -- | 0.12 | 1.00 | -- | -- |
|  | **NMFS** | 0.10 | 0.53 | 0.05 | 7.09 | 0.06 | 0.42 | 0.53 | 6.52 | 0.09 | 0.49 | 0.05 | 7.26 | 0.56 | 0.79 | 0.11 | 8.90 | 0.09 | 0.52 | 0.05 | 6.36 |
|  | **2Tmin** | 0.05 | 0.61 | 1.38 | 4.70 | 0.03 | 0.53 | 1.90 | 4.67 | 0.06 | 0.60 | 1.49 | 4.50 | 0.49 | 0.82 | 1.62 | 9.03 | 0.07 | 0.60 | 1.60 | 4.65 |
|  | **.75FMSY** | 0.02 | 0.56 | 1.44 | 5.34 | 0.02 | 0.50 | 1.80 | 5.08 | 0.03 | 0.58 | 1.58 | 5.02 | 0.36 | 0.84 | 3.48 | 7.46 | 0.04 | 0.50 | 1.71 | 5.13 |
|  | **4010** | 0.12 | 0.65 | 0.03 | 6.61 | 0.11 | 0.56 | 0.03 | 6.13 | 0.10 | 0.68 | 0.03 | 6.52 | 0.66 | 0.92 | 0.61 | 8.63 | 0.12 | 0.69 | 0.03 | 5.88 |
|  |  |  |  |  |  |  |  |  |  |  |  |  |  |  |  |  |  |  |  |  |  |
| **Rockfish** | **NoFish** | 0.00 | 0.76 | -- | -- | 0.00 | 0.79 | -- | -- | 0.00 | 0.79 | -- | -- | 0.41 | 1.00 | -- | -- | 0.01 | 0.79 | -- | -- |
|  | **NMFS** | 0.00 | 0.38 | 0.47 | 12.53 | 0.00 | 0.53 | 0.55 | 8.04 | 0.00 | 0.36 | 0.45 | 5.97 | 0.40 | 0.60 | 0.94 | 50.80 | 0.01 | 0.12 | 0.52 | 22.08 |
|  | **2Tmin** | 0.00 | 0.32 | 0.69 | 22.32 | 0.00 | 0.42 | 2.91 | 17.46 | 0.00 | 0.32 | 0.71 | 22.49 | 0.39 | 0.59 | 0.98 | 51.27 | 0.01 | 0.11 | 1.84 | 23.07 |
|  | **.75FMSY** | 0.00 | 0.26 | 7.26 | 23.80 | 0.00 | 0.25 | 8.37 | 23.09 | 0.00 | 0.25 | 7.47 | 23.56 | 0.24 | 0.53 | 13.26 | 42.46 | 0.00 | 0.08 | 8.91 | 26.75 |
|  | **4010** | 0.00 | 0.46 | 0.38 | 21.03 | 0.00 | 0.44 | 0.38 | 22.91 | 0.00 | 0.44 | 0.38 | 20.12 | 0.39 | 0.64 | 1.46 | 42.22 | 0.01 | 0.25 | 0.39 | 32.79 |
|  |  |  |  |  |  |  |  |  |  |  |  |  |  |  |  |  |  |  |  |  |  |
