# Supplementary material for: An Evaluation of Rebuilding Policies for U.S. Fisheries
Source: PLoS One. 2016 Jan 13;11(1):e0146278. doi: 10.1371/journal.pone.0146278 (PMC4711967; doi:10.1371/journal.pone.0146278)
Supplement: S3 Table — (DOCX) [file pone.0146278.s004.docx]

S3 Table.

|  | **Baseline** | | | **High *M*** | | | **MGT** | | | **Initial *D*** | | | **2 Fleet** | | |
| --- | --- | --- | --- | --- | --- | --- | --- | --- | --- | --- | --- | --- | --- | --- | --- |
|  | **2TMIN** | **4010** | **0.75*F_MSY_*** | **2TMIN** | **4010** | **0.75*F_MSY_*** | **2TMIN** | **4010** | **0.75*F_MSY_*** | **2TMIN** | **4010** | **0.75*F_MSY_*** | **2TMIN** | **4010** | **0.75*F_MSY_*** |
| **Alternative** | |  |  |  |  |  |  |  |  |  |  |  |  |  |  |
| Mackerel | 0.48 | 0.86 | 0.51 | 0.56 | 0.79 | 0.68 | 0.56 | 0.84 | 0.62 | 0.73 | 0.89 | 0.49 | -- | -- | -- |
| Butterfish | 0.95 | 0.98 | 0.94 | 0.96 | 0.98 | 0.96 | 0.95 | 0.99 | 0.94 | 0.98 | 0.99 | 0.97 | -- | -- | -- |
| Sole | 0.15 | 0.96 | 0.14 | 0.16 | 0.93 | 0.33 | 0.11 | 0.97 | 0.13 | 0.74 | 0.97 | 0.44 | -- | -- | -- |
| Snapper | 0.53 | 0.95 | 0.25 | 0.51 | 0.84 | 0.24 | 0.52 | 0.93 | 0.24 | 0.91 | 0.84 | 0.35 | 0.60 | 0.97 | 0.21 |
| Porgy | 0.57 | 0.89 | 0.56 | 0.62 | 0.91 | 0.70 | 0.59 | 0.86 | 0.59 | 0.81 | 0.95 | 0.46 | 0.58 | 0.90 | 0.54 |
| Rockfish | 0.53 | 0.78 | 0.41 | 0.39 | 0.60 | 0.20 | 0.56 | 0.73 | 0.46 | 0.86 | 0.79 | 0.31 | 0.64 | 0.92 | 0.65 |
|  |  |  |  |  |  |  |  |  |  |  |  |  |  |  |  |
| **NMFS** | |  |  |  |  |  |  |  |  |  |  |  |  |  |  |
| Mackerel | 0.68 | 0.38 | 0.57 | 0.56 | 0.33 | 0.42 | 0.61 | 0.35 | 0.49 | 0.77 | 0.50 | 0.66 | -- | -- | -- |
| Butterfish | 0.94 | 0.58 | 0.67 | 0.95 | 0.65 | 0.64 | 0.94 | 0.59 | 0.60 | 0.96 | 0.77 | 0.74 | -- | -- | -- |
| Sole | 0.95 | 0.46 | 0.90 | 0.90 | 0.30 | 0.74 | 0.97 | 0.41 | 0.91 | 0.95 | 0.44 | 0.85 | -- | -- | -- |
| Snapper | 0.79 | 0.44 | 0.81 | 0.78 | 0.47 | 0.79 | 0.77 | 0.61 | 0.79 | 0.92 | 0.80 | 0.93 | 0.83 | 0.52 | 0.83 |
| Porgy | 0.54 | 0.38 | 0.47 | 0.43 | 0.22 | 0.34 | 0.52 | 0.32 | 0.46 | 0.83 | 0.45 | 0.68 | 0.55 | 0.32 | 0.47 |
| Rockfish | 0.57 | 0.36 | 0.61 | 0.69 | 0.54 | 0.81 | 0.50 | 0.45 | 0.55 | 0.92 | 0.75 | 0.74 | 0.48 | 0.23 | 0.35 |
|  |  |  |  |  |  |  |  |  |  |  |  |  |  |  |  |
|  |  |  |  |  |  |  |  |  |  |  |  |  |  |  |  |
|  |  |  |  |  |  |  |  |  |  |  |  |  |  |  |  |
