# Supplementary material for: An Evaluation of Rebuilding Policies for U.S. Fisheries
Source: PLoS One. 2016 Jan 13;11(1):e0146278. doi: 10.1371/journal.pone.0146278 (PMC4711967; doi:10.1371/journal.pone.0146278)
Supplement: S4 Table — Compare to S1 Table. (DOCX) [file pone.0146278.s005.docx]

S4 Table.

|  | **NMFS Faster** | **Equal** | **2TMIN Faster** |
| --- | --- | --- | --- |
| Mackerel | 0.27 | 0.50 | 0.23 |
| Butterfish | 0.02 | 0.93 | 0.04 |
| Snapper | 0.09 | 0.83 | 0.08 |
| Porgy | 0.19 | 0.64 | 0.17 |
| Sole | 0.26 | 0.70 | 0.05 |
| Rockfish | 0.14 | 0.78 | 0.08 |
|  |  |  |  |
|  | **NMFS Shorter** | **Equal** | **40-10 Faster** |
| Mackerel | 0.11 | 0.38 | 0.50 |
| Butterfish | 0.01 | 0.76 | 0.23 |
| Snapper | 0.17 | 0.64 | 0.20 |
| Porgy | 0.05 | 0.40 | 0.55 |
| Sole | 0.04 | 0.41 | 0.56 |
| Rockfish | 0.21 | 0.53 | 0.25 |
|  |  |  |  |
|  | **NMFS Shorter** | **Equal** | **0.75Fmsy Faster** |
| Mackerel | 0.51 | 0.16 | 0.34 |
| Butterfish | 0.03 | 0.72 | 0.26 |
| Snapper | 0.66 | 0.27 | 0.08 |
| Porgy | 0.54 | 0.13 | 0.32 |
| Sole | 0.57 | 0.29 | 0.15 |
| Rockfish | 0.69 | 0.05 | 0.26 |
